# Role of Biomarkers in the Integrated Management of Melanoma

**DOI:** 10.1155/2021/6238317

**Published:** 2021-12-30

**Authors:** Piyu Parth Naik

**Affiliations:** Department of Dermatology, Saudi German Hospital and Clinic, Opposite Burj Al Arab, Dubai, UAE

## Abstract

Melanoma, which is an aggressive skin cancer, is currently the fifth and seventh most common cancer in men and women, respectively. The American Cancer Society reported that approximately 106,110 new cases of melanoma were diagnosed in the United States in 2021, with 7,180 people dying from the disease. This information could facilitate the early detection of possible metastatic lesions and the development of novel therapeutic techniques for melanoma. Additionally, early detection of malignant melanoma remains an objective of melanoma research. Recently, melanoma treatment has substantially improved, given the availability of targeted treatments and immunotherapy. These developments have highlighted the significance of identifying biomarkers for prognosis and predicting therapy response. Biomarkers included tissue protein expression, circulating DNA detection, and genetic alterations in cancer cells. Improved diagnostic and prognostic biomarkers are becoming increasingly relevant in melanoma treatment, with the development of newer and more targeted treatments. Here, the author discusses the aspects of biomarkers in the real-time management of patients with melanoma.

## 1. Introduction

Melanoma is an increasingly prevalent, potentially fatal skin cancer. Melanoma can develop on the leptomeninges, mucosal surfaces, and the uveal tract, with cutaneous melanoma being the most prevalent type [1, 2]. Melanoma was once considered uncommon; however, its prevalence has increased faster than any other cancer within the previous 50 years [3]. In 2017, melanoma was detected in over 85,000 new instances in the United States (US), which makes it the sixth most prevalent cancer, with >9500 deaths [4]. Among skin cancer, melanoma accounts for the highest mortality but accounts for >5% of all cutaneous malignancies [3]. Melanoma originates in cutaneous melanocytes and is classified as noncutaneous or cutaneous. Cutaneous melanoma has four primary subtypes: acral lentiginous (<5%), lentigo maligna melanoma (4%–10%), nodular melanoma (15%–30%), and superficial spreading melanoma (70%) [5]. Melanoma can develop in any noncutaneous site with melanocytes, including the nasopharyngeal, genitourinary, ocular, and gastrointestinal regions. Melanoma of unknown primary location, ocular melanoma, and mucosal melanoma account for 2.2%, 5.2%, and 1.3%, respectively [2, 6]. Melanoma prognosis is determined by lesion's thickness, with thicker lesions indicating a higher mortality rate [7]. Absolute abscission of melanoma causes exceptional emanation in the early stages. However, melanoma is a dangerous cancer that often spreads beyond its primary site. Surgery cannot comprehensively manage advanced melanoma, with the disease becoming more challenging to treat [3, 8]. Despite recent advances in metastatic melanoma therapy, melanoma with distant metastases still has a poor prognosis, with a 5-year survival rate of only 16% [9]. Since patients with late-stage melanoma have a poor prognosis, biomarkers are necessary for treatment and identifying individuals requiring aggressive treatment. These biological markers facilitate melanoma diagnosis and monitoring of melanoma recurrence after surgical resection and the influence of radiation or anticancer medication therapy. Accordingly, the current review discusses the potential role of biomarkers in the integrated management of melanoma.

## 2. Methodology

**Figure 1:**
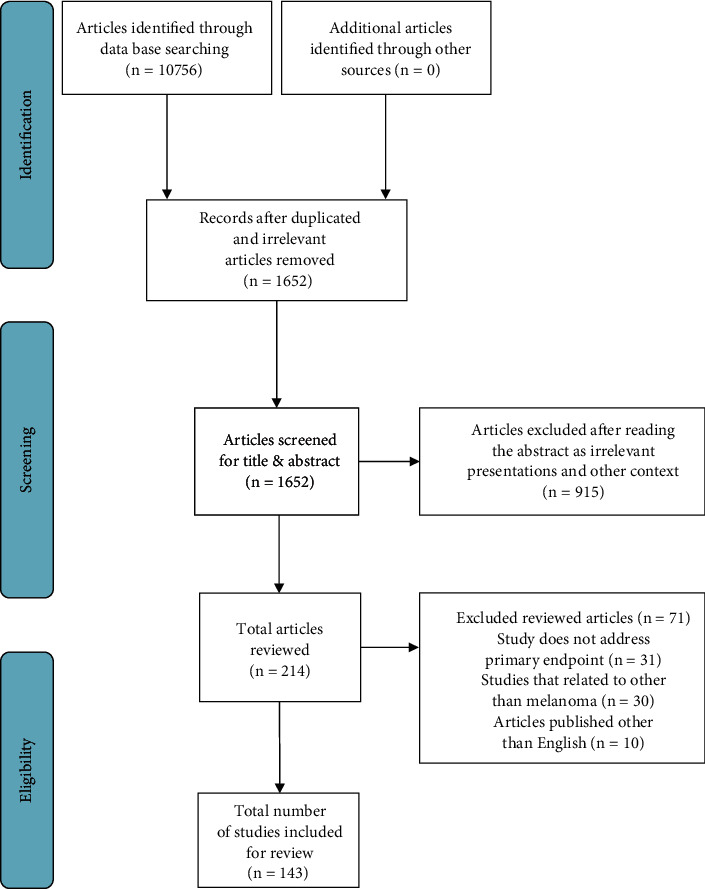
PRISMA flow chart.

The following terms were combined to search MEDLINE and PubMed: Biomarkers AND “melanoma” OR “stem cell “OR “melanoma” AND “cyclooxygenase” OR “biomarker”) AND (“skin neoplasms” OR (“skin” AND “neoplasms”) OR “skin neoplasms” OR (“skin” AND “cancer”) OR “skin cancer”) AND (“melanoma” OR “melanoma”) AND (“lactate dehydrogenase” OR “diagnosis” OR “screening” OR “tyrosinase” OR (“S100” AND “screening”) OR AND (“diagnosis” OR “diagnosis” OR “screening” OR “mass screening” OR (“mass” AND “screening”) OR “mass screening” OR “screening” OR “early detection of cancer” OR (“early” AND “detection” AND “cancer”) OR “early detection of cancer”) AND (“early diagnosis” OR (“early” AND “diagnosis”) OR “early diagnosis”). Primarily, the author searched for articles regarding biomarkers and their role in managing melanoma. As shown in Figure 1, the initial literature search revealed 10756 articles. This review included articles published in English between January 1990 and July 2021 that described biomarkers in melanoma management.

## 3. Biomarkers Associated with the Treatment of Melanoma

Over the past few decades, there has been a progressive increase in the global incidence of melanoma. In many fair-skinned communities, the prevalence has increased to 4%–6%, especially in Australia, New Zealand, Northern Europe, and North America. Additionally, there has been a significant increase in the incidence rates in communities of many ethnicities and geographic locations and within populations of different ages and genders [3]. Therefore, the basic concept of melanoma control is therapy personalization; high-risk groups should be diagnosed early and sensitive. In this context, biomarkers represent unique molecular characteristics of a patient that allow the detection and diagnosis of cancer with respect to tumor's biological behavior, sensitivity to therapy, or resistance mechanisms. Biomarkers may significantly improve melanoma therapy and facilitate simple detection. Moreover, they could allow identification and treatment of melanoma before it becomes apparent or symptomatic, as well as screening according to the general population.

**Figure 2:**
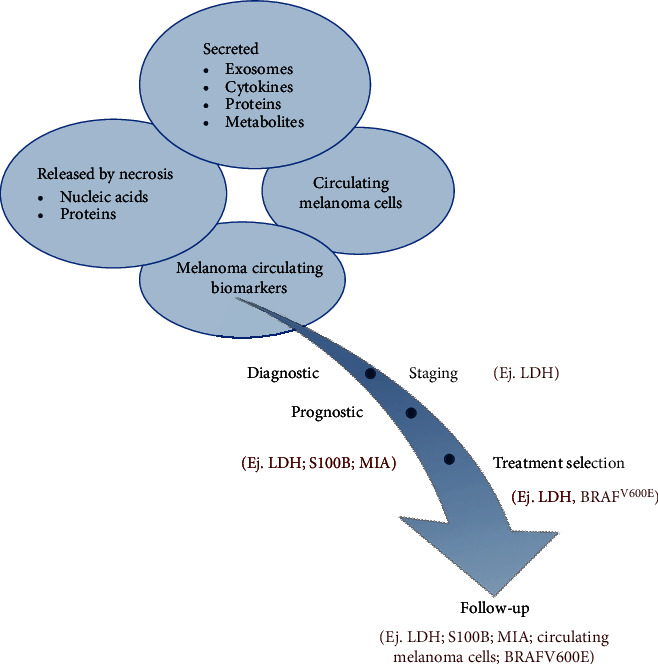
Production of different types of tumor markers by melanoma cells and their potential utility.

Melanoma biomarkers can be classified into several groups. They are utilized as diagnostic markers since they are increased in tumor cells than in normal tissue compared to healthy participants. Several biomarkers have prognostic or predictive utility given their amplification in the late phases of cutaneous melanoma during therapeutic repercussions or disease reappearance indicators during the follow-up period [10].  Figure 2 presents the formulation of numerous biomarkers in tumor cells, as well as their prepatent pertinence. Melanoma cells release several molecules and proteins into the extracellular fluid. Some of these molecules can enter the bloodstream and be used as serum biomarkers. These biomarkers comprise molecules, including enzymes, circulating cell-free nucleic acids, antigens or soluble proteins, and melanin-related metabolites. From a pathobiochemical perspective, they are released through ectodomain membrane shedding, necrosis, and active secretion [11]. These molecules have varying prognosticative merits in treatment monitoring and melanoma diagnosis [12, 13]. Nonetheless, biomarkers determined through immunohistochemical and histological assays of biopsy substances have indispensable applicability in melanoma therapy.  Table 1 lists the potential biomarkers for malignant melanoma.

## 4. Commonly Used Methodologies for the Detection of Melanoma Biomarkers

The current gold standard for a melanoma diagnosis is the histologic interpretation of cytomorphologic and architectural characteristics, which is still one of the most challenging areas of dermatopathology practice. This inherently and inevitably subjective practice is subject to well-documented pathologist interobserver and intraobserver variability [34–36] and diagnostic drift [37].

### 4.1. myPath Melanoma: 23-Gene Expression Profiling (Myriad Genetics)

The myPath Melanoma diagnostic test from Myriad Genetics can help distinguish between benign melanocytic nevi and malignant melanoma. This assay, like the DecisionDx-Melanoma test, uses reverse transcriptase-polymerase chain reaction technology, but instead of evaluating the expression of 23 genes, it uses an algorithm that assigns different weights and expression thresholds to each gene [38–40].

### 4.2. Pigmented Lesion Assay: 2-Gene Expression Profiling (DermTech)

Molecular testing has made its way from pathology laboratories to dermatology clinics to aid clinicians in their biopsy decisions. DermTech recently published research on the development, validation, and clinical utility of its noninvasive adhesive patch “skin biopsy,” which collects 1.5 mg of the stratum corneum tissue containing approximately 23 ng of human skin RNA from the underlying melanocytic neoplasm, as well as human skin DNA, microbial DNA, proteins, lipids, and sugars [41, 42].

### 4.3. DecisionDx-Melanoma: 31-Gene Expression Profiling (Castle Biosciences)

The DecisionDx-Melanoma test from Castle Biosciences assesses the risk of metastasis in cases where melanoma has previously been diagnosed. This assay uses a reverse transcriptase-polymerase chain reaction approach to create a messenger RNA-based gene expression profile assay that includes 28 prognostically essential genes and three control genes [43].

### 4.4. FISH Testing

FISH is a molecular technique that detects complementary genomic DNA sequences on metaphase and/or interphase nuclei in tissue sections using fluorescent DNA locus-specific probes, enabling for direct viewing of specific genomic DNA segments. For melanocytic lesions, there are two types of probes: centromere probes, which identify centromeric areas on chromosomes, and locus-specific probes, which hybridize onto target sequences spanning genes or regions of interest. FISH assays can identify chromosomal deletions, amplifications, and translocations depending on the probe(s) used [44].

### 4.5. qRT-PCR

qRT-PCR is a molecular biology technique for detecting the amount of expression of specific RNA transcripts. In brief, the technique entails transcribing RNA to complementary DNA (cDNA) and then performing real-time PCR. Transcriptome data from extensive-expression array experiments can be analyzed for substantial changes in RNA expression between neoplasms and used to create gene expression signatures that distinguish between benign and malignant tumors [45].

### 4.6. 5-Hydroxymethylcytosine

Finally, immunohistochemical detection of loss of the epigenetically changed DNA base 5-hydroxymethylcytosine (5-hmC) is a potentially valuable diagnostic and prognostic adjunct in assessing melanocytic proliferations [46]. 5-hmC is a crucial step in the teneleven translocase 2- (TET2-) mediated DNA demethylation process, vital for DNA damage detection, telomere maintenance, and genomic stability [47, 48]. 5-hmC loss has been proven to be a diagnostic feature of malignant melanoma and discriminate between a spectrum of histologic and genetically diverse melanoma subtypes and benign nevi with high sensitivity and specificity [49–54].

## 5. Prognostic Biomarkers

The broadest category of biomarkers is prognostic biomarkers. They range from predictors of survival prospects to those of probable melanoma return. The main limitation of survival indicators is that they might require adoption by the American Joint Committee on Cancer (AJCC) or other similar staging protocols before being widely used. Otherwise, the requesting clinician would be confused about how an adverse outcome alters patient's clinical-stage, intervention options, and eventual outcomes. A biomarker with good test features should be integrated into a staging groundwork for clinical decision-making to allow utility. The Castle DecisionDx assay is a commercially available prognostic and diagnostic tool. Although it is not included in the AJCC staging or National Comprehensive Cancer Network treatment guidelines, it can identify patients with stage I and II cancer at a higher risk of metastasis and mortality. The gene expression profile has shown consistency across analytical instruments in differentiating “class 1” (low-risk) from “class 2” (high-risk) probability scores, as well as technical concordance between runs [55]. Small-scale retrospective and prospective clinical cohorts have improved stratification of the risk of relapse and distant metastases regardless of the sentinel lymph node biopsy status [56–60]. Applying both DecisionDx probability scores and AJCC stages (“low-risk”: IB-IIA, “high-risk”: IIBIIC), Podlipnik et al. reported a lower disease-free survival (DFS) in patients with melanoma with DecisionDx high-risk “class 2” scores, regardless of the AJCC staging [60].

Ki-67 is a nuclear antigen expressed throughout the cell cycle's active phases and is a proliferation marker (G1, S, G2, and M) [61]. The ki-67 expression has been shown to connect directly with prognosis in thin melanomas (less than 1 mm) and may link more strongly with prognosis than mitotic count [62, 63]. Furthermore, in thicker melanomas (>1 mm), Ki-67 has been demonstrated to be superior to mitotic count as a predictive indicator for survival [64]. Furthermore, histopathologists' detection of mitoses has a considerable degree of interobserver heterogeneity [65].

Sentinel lymph node involvement is more likely in tumors with greater mitotic rates, Breslow thickness, and the absence of tumor-infiltrating lymphocytes [66]. The number of nodal metastases is the single most important predictor of patient survival in patients with stage III cancer; therefore, these markers are even more critical [67].

More than half of all melanomas have BRAF mutations, with the V600E mutation accounting for nearly all [68, 69]. The MAPK pathway can be activated indefinitely if specific mutations occur [70]. BRAF mutations, particularly the V600E mutant, have not been linked to a substantial difference in patient survival compared to melanomas that do not contain this mutation [71]. However, in individuals with late-stage melanoma who carry the V600E mutation, the BRAF inhibitor vemurafenib has been proven to improve survival. As a result of this new treatment, patients with tumors positive for the BRAF mutation may have a better prognosis [72].

MCAM, also known as MUC18 or CD146, is a 113-kDa cell adhesion protein customarily expressed on endothelium and smooth muscle cells in adult tissue [73]. It is substantially expressed in advanced primary and metastatic melanoma and less so in nevi, even though it is rarely expressed in carcinomas [74–76]. MCAM expression is an independent predictor of outcome [77, 78].

## 6. Stem Cell-Like Markers

Other than the aforementioned indicators, animal studies have identified auxiliary proteins in circulating melanoma cells. Few could be markers for melanoma progenitor cells or stem cells, including the neuroepithelial intermediate filament nestin and ATP-binding cassette multidrug transporters [11, 13]. Hong and Saint-Jeannet reported a positive relationship between nestin expression and advanced disease in several melanoma specimens [79]; however, they observed high nestin expression in compound nevi. Notably, progenitor cells include Sry-related HMG-box (SOX) proteins. In some cases, nuclear transcription factors are involved in developing neural crest progenitor cells into melanocytes; however, supplements are more resourceful fate regulators of stem and progenitor cells [79, 80].

The immunohistochemistry profile of SOX10 is a reliable marker for diagnosing metastatic melanoma in sentinel lymph nodes, with increased specificity and sensitivity when combined with additional immunohistochemical stains, including melan A or S100B [14]. However, SOX10 staining cannot distinguish melanoma metastases from nodal nevi [14]. Contrastingly, in addition to nestin, SOX2 can successfully distinguish nodal melanocytic nevi from metastatic melanoma and could be a diagnostic tool in melanoma staging [15]. Variations in the biomarker utility of multiple SOX protein family members are consistent with the extensive heterogeneity of melanoma. Numerous other biomarkers were in the same position. These previous inconsistent findings essentially confound the final assessment. For example, the significance of two other stem cell-like markers, CD133 (*syn*. prominin-1) and CD271 (nerve growth factor receptor), which have recently been identified as critical molecules that promote melanoma initiation and metastasis, remains unclear [81–83].  Table 1 lists other proteins that could be used as candidate melanoma biomarkers. Furthermore, melanoma biomarker research could focus on nonprotein biomarkers, including the metabolites of the melanin production pathway derived from cell-free nucleic acids and the amino acid l-tyrosine [11].

## 7. Inhibitors of Endogenous Enzymes

Tissue inhibitors of metalloproteinases (TIMPs) such as TIMP-1, which are endogenous inhibitors of MMPs, are important in tumor formation. TIMPs are involved in differentiation, apoptosis, angiogenesis, extracellular matrix degradation, and proliferation of normal and malignant cells [84]. Compared with healthy individuals, patients with stage I–III melanoma have higher median serum TIMP-1 levels, influencing overall and DFS. However, there is no association between invasion depth and TIMP-1 levels, clinical stage, or nodal status with respect to MMP-9 [27]. SerpinB1 has been proposed as a marker for the chemotherapeutic response. Willmes et al. reported clinical and experimental data regarding serpinB1 expression, which indicates that cisplatin-based therapy regimens could help patients with stage IV cutaneous melanoma with high serpinB1 protein amplification.

Furthermore, serpinB1 protein expression can predict the outcome of melanoma chemotherapy with cisplatin [85]. There has been increasing attention to other protease inhibitors, including serpinB1 and maspin (serpinB5), serine protease inhibitor superfamily members. Maspin loss in melanoma could contribute to the metastatic spread and disease progression; however, this remains unclear [86, 87].

## 8. Cyclooxygenase-2

Cyclooxygenases act as significant modulators in the human body and influence crucial processes, including catabolic metabolism. Cyclooxygenases convert arachidonic acid to prostaglandins. Cyclooxygenase-2 is activated in tumor cells [88, 89]. In melanoma, Becker et al. reported an association of the staining magnitude of cyclooxygenase-2 with Breslow depth [90]. Additionally, Kużbicki et al. found that compared with benign nevi, melanoma lesions had a higher cyclooxygenase-2 staining intensity [91]. Several studies have reported that cyclooxygenase-2 is a potential immunohistochemical marker in the oral cavity for differentiating between melanoma and benign melanocytic lesions [92]. These findings suggest that cyclooxygenase-2 expression plays a pathogenic role in melanoma and is a prospective molecular target [93]. Kużbicki et al. developed an immunohistochemical scoring methodology, which demonstrates the significance of cyclooxygenase-2 as a negative prognostic factor for melanoma, directly associated with other relevant prognostic variables, including ulceration, lymph node metastasis, and tumor thickness [94]. Hennequart et al. reported that cyclooxygenase-2 shaped the immunosuppressive tumor microenvironment in nonmelanoma and melanoma (KUL98-MELA) cell line tumors. Recent studies have synthesized new celecoxib analogs with significant cytostatic activity against melanoma cells [95]. Recent studies have synthesized new celecoxib analogs with significant cytostatic activity against melanoma cells [96]. An ongoing phase II trial is assessing the antiproliferative utility of aspirin combined with CTLA4 (ipilimumab) and PD-1 (pembrolizumab) inhibitors [97]. These findings provide insight into the efficacy of nonsteroidal anti-inflammatory drugs in melanoma treatment.

## 9. Lactate Dehydrogenase

LDH is primarily secreted in response to cell death or injury, indicating increased tumor burden and disease progression. However, increased serum LDH levels are not limited to cancer and can occur with inflammation, infarction, infection, and hemolysis. Consequently, the false-positive rate restricts the positive predictive value in melanoma [10]. Recent studies have demonstrated that LDH is imperceptive in early-stage melanoma but has a negative prognostic value for metastatic relapse [98–100].

There have been recent studies on serum LDH as a prognostic indicator in patients with advanced melanoma treated through immunomodulatory drugs. In patients with metastatic melanoma treated with ipilimumab, the baseline serum LDH level is a strong predictor of overall survival (OS) [101]. Specifically, patients with a serum LDH level that is more than twice the upper standard limit at baseline are unlikely to benefit from long-term ipilimumab treatment. Another study reported that low serum LDH is associated with a positive outcome in patients with late-stage melanoma treated with ipilimumab, which confirms that baseline serum LDH could guide prognosis in patients with advanced melanoma [102]. There has been a recent further establishment of the importance of LDH as a predictor and measure of therapeutic response. A meta-analysis study reported an association of high blood LDH levels with shorter OS in patients with melanoma [103]. According to an independent investigation, low baseline serum LDH is related to a positive result in patients with late-stage melanoma treated with ipilimumab, confirming that baseline serum LDH is a potential marker for prognosis in patients with advanced melanoma [102]. Fischer et al. reported that elevated serum LDH is associated with poor clinical outcomes in stage IV metastatic melanoma patients. Using various thresholds (≥3, *p* < 0.0001; ≥4, *p* = 0.0011; ≥5, *p* = 0.0038), univariate analysis revealed that patients with elevated serum LDH (*n* = 34) had a substantially higher number of sites with metastatic involvement than patients without elevated serum LDH (*n* = 75) [104].

## 10. Matrix Metalloproteinases

Matrix metalloproteinases are necessary for proteolytic splicing of the sectarian tissue framework, which allows tumor cell migration and promotes tissue remodeling, contributing to modifying the tumor tissue microenvironment [105, 106]. These factors lead to protein overexpression in tumor tissues. Nikkola et al. observed that MMP-1- and MMP-3-positive melanoma metastases are associated with a shorter DFS [107]. Furthermore, Rotte et al. reported that compared with normal and dysplastic nevi, melanoma has greater MMP-2 expression; moreover, MMP-2 expression was strongly associated with negative tumor evolution and poor survival [32]. However, it is essential to note that Rotte et al. used tumor tissue microarrays and peroxidase produced using 3,3′-diaminobenzidine; therefore, MMP-2 measurement in pigmented lesions could be limited in distinguishing melanoma cells.

Furthermore, there have been recent studies on MMPs as potential melanoma biomarkers and immunotherapeutic targets. For example, the MMP-2 activator, MT1-MMP, has a higher expression in early melanoma than in nevi; moreover, it continues to increase with disease progression, indicating poor patient outcomes [29]. Additionally, MMP-12 is strongly associated with invasion and metastasis in cutaneous melanoma compared with normal skin. Additionally, patients with high MMP-12 levels have poor OS [28].

Other proteins closely regulate MMP activity, ensuring steady-state conditions between ECM's degradative and reconstructive processes [108]. MMPs are regulated by a type of endogenous inhibitor known as TIMP, which has a role in activating and inactivating MMPs [109]. The numerous structures that characterize MMPs allow them to perform multiple roles that influence various processes such as cell behavior, apoptosis, and cell proliferation. MMPs have been found to promote tumor progression by degrading surrounding tissues, modulating growth factors and membrane receptors, as well as inflammatory proteins, adhesion molecules, and chemo-attractive proteins, according to oncological studies [110–112]. MMPs are also involved in the alteration of the ECM of the skin. MMPs play a role in skin matrix remodeling by degrading and reconstructing matrix components. In addition, multiple studies have shown that MMPs play a critical role in melanoma, with tumor cells and tumor microenvironment changes linked to MMP and TIMP deregulation [113].

MMP inhibitors (MMPi) are substances that interact with MMPs to modulate their actions. There are two types of MMP inhibitors: synthetic inhibitors and endogenous inhibitors [114]. Since the 1990s, pharmaceutical companies have been developing MMPi medicines to treat diseases in which proteinases are dysregulated, such as cancer. Marimastat (BB-2516) and Cipemastat (Ro 32-3555) were the first, both containing the hydroxamate group [115]. Chirvi et al. (1994) investigated the efficacy of Batimastat, a first-generation MMP inhibitor, in C57BL/6N mice injected with B16-BL6 melanoma cells to assess tumor growth inhibition. The results demonstrated a reduction in lung metastasis and solid tumor dimensions when the medicine was administrated after inoculation [116]. Another in vivo investigation was conducted on a melanoma animal model to determine the efficacy of Batimastat when combined with IL12; the results demonstrated substantial antitumoral and antiangiogenic effects in this scenario [117].

The preferred contemporary path for treating advanced melanoma patients is utilizing an amalgamation of medicines that impede the kinase actions of BRAF and anti-MEK. Dabrafenib, a BRAF inhibitor, is employed in specific BRAF^V600E^-mutated metastatic or advanced melanoma patients. Similarly, Trametinib, a MEK-1/2 inhibitor, is also available in metastatic or advanced cases with BRAF^V600E^ mutation. These therapeutic boulevards have paved the way for recognizing potential biomarkers which ultimately can be utilized to assess the therapeutic response of these treatments. Recent research has focused on identifying circulating-free (cfDNA) BRAF^V600E^ mutation in patients treated with BRAF/MEK inhibitors. Circulating-free (cfDNA) BRAF^V600E^ mutation was linked to a bad prognosis. Also, a positive correlation betwixt circulating-free (cfDNA) BRAF mutation and MMP-protein was detected. This has emphasized the roles of MMPs and cfDNA in establishing resistance or response to treatment [118–120].

## 11. Tyrosinase

Tyrosinase is a melanin-producing enzyme found in melanocytes and melanoma cells. Nested reverse transcription-polymerase chain reaction (RT-PCR) can detect tyrosinase mRNA grades in the blood samples of patients with melanoma and late-stage metastatic illness. Preliminary studies have indicated that tyrosinase is a sovereign predictive pathfinder for tumor progression [121, 122]. Šamija et al. reported that tyrosine mRNA is associated with reduced OS [123]. Salvianti et al. studied the diagnostic relevance of hypermethylated *Ras* association domain family one isoform A promoter as a neoplasm-related, methylated, and cell-free DNA marker [124]. This marker can distinguish between healthy controls and patients with melanoma.

Moreover, tyrosinase has been considered a tissue biomarker. Lin et al. published a novel approach for mapping the distribution and expression of type 3 copper protein tyrosinase on tissue microarrays of skin samples of patients with melanoma using scanning electrochemical microscopy [125]. The transition from a homogeneous tyrosinase distribution in stage II to a more heterogeneous pattern in stage III was visualized. Notably, optically interfering species, including melanin, do not hinder scanning electrochemical microscopy. This marker could allow the diagnosis of nonmetastatic and metastatic melanoma stages as a complementary prognostic technique.

## 12. Other Enzyme Markers

Studies currently investigate other potential melanoma enzyme markers, including CD10, legumain, and proteases cathepsin K [25, 26, 30]. However, it is difficult to determine their utility as biomarkers given the scarce research. Aldehyde dehydrogenase 1 (ALDH-1) is a potential therapeutic target and biomarker of stem cells in several human neoplasms, including melanoma [126, 127]. According to Taylor et al., ALDH-1 could be an independent prognostic factor in melanoma [128].

## 13. S100 Proteins

Since the secretion and expression of S100 proteins are significantly higher in cancerous tissues than in normal tissues, the S100 protein family has become a distinct diagnostic indicator in cutaneous melanoma throughout the last decade [129–131]. Patients with melanoma have increased serum levels of S100 proteins, especially S100B, with higher strata linked to a worse prognosis [132, 133], DFS, and OS [134]. S100B was associated with lower OS and distant metastasis-free survival in a contemporary prognostic assessment of patients treated with IFN-*α*2b than stage II and III patients. Serum S100B levels correspond to disease development throughout time; moreover, they increase with disease severity [135]. False-positive results can result from harm to the cerebral parenchyma, hepatic region, or kidney and during infections [136–138].

Wevers et al. reported a strong correlation of S100B levels with melanoma prognosis in stage IIIB–IIIC patients. Preoperative and postoperative (day 2) S100B readings were strongly correlated with DFS. S100B levels could be the most significant independent predictor of disease-specific survival [139]. Increased S100B protein expression has been observed in 74%–100% of patients with stage IV melanoma [140, 141]. Several studies have reported a positive correlation between DFS and advanced disease stage [16, 141, 142]. S100A8/A9 is a unique predictive guide for ipilimumab treatment in metastatic stage IV melanoma patients. Nonresponding and responding patients with melanoma significantly increased and decreased S100A8/A9 serum levels after the first ipilimumab infusion [22]. Skaleinius [143] reported that patients with a high baseline S100 had a significantly shorter OS (*p* = 0.0038) than those with a normal S100. The aforementioned analyses were performed on 41.4% of individuals with a high baseline S100. However, there remains a need for further research on S100 as a possible clinical tool [143].

## 14. Immunotherapy and Biomarkers

Targeted immunotherapy in patients with melanoma depends on precise characters of neoplasm. Circulating-free (cfDNA) BRAF mutation and MMP-protein detection processes have attempted to detect exact prognoses in those cases. However, the lack of biomarker response in patients with immune checkpoint inhibitor combinations is a real challenge. In patients with PD-1/PD-1 inhibitors, immunohistochemistry(IHC) staining can be employed to determine the PD-L1 expression [144]. A recent study in anti-PD-1 responding cases detected novel links between IL-6, IL-10, desmocollin 3, proline-rich acidic protein 1, C-C motif chemokine ligands, vascular endothelial growth factor, and progression-free survival. Elevated circulating PD-1 was also demonstrated in this landmark study. This milestone research has described the capability of plasma proteomics as a liquid biopsy approach. Identifying these protein biomarkers has laid the foundation of future research in patients of metastatic cutaneous melanoma as they can be pivotal to defining the prognosis for anti-PD-1 treatment [145].

## 15. Conclusion

Several tissue and serum biomarkers can predict overall survival and disease progression. There is current research for identifying, establishing, and validating the best biomarker combination for multimarker profiling in patients with melanoma. Proteomic profiling studies could identify melanoma-specific markers for improved prognosis. Furthermore, identifying biomarkers that predict response to medicines, especially new immunotherapies, is the next step in melanoma biomarker research, which will allow clinicians to choose the best treatment option.

## Figures and Tables

**Table 1:** Potential biomarkers in malignant melanoma.

	Major laboratory methodologies	Correspondence with	Biomarkers	References
Nucleic acids	RT-PCR	Overall survival	miRNA-29c	[11]
	RT-PCR	*Breslow index*	miRNA-221	[11]
Metabolites∗	HPLC	*Breslow index*	6H5MI2C	[11]
	HPLC	Poor prognosis, response to treatment	5-S-cysteinyl-DOPA	[11]
	HPLC	Tumor burden, tumor progression	l-DOPA/l-tyrosine	[11]
Progenitor/stem cell-like markers	IHC	Disease	SOX protein family	[14, 15]
S100 proteins	ELISA, LIA	Tumor stage, survival, recurrence	S100B	[16, 17]
	MS, IHC	Tumor progression	S100A13	[18, 19]
	Northern blot	Tumor progression (negative correlation)	S100A2	[11]
	IHC	Tumor progression	S100P	[11, 20]
	IHC, ELISA, FC	Tumor progression	S100A8/A9	[21, 22]
	IHC	Tumor progression	S100A4	[11]
	Northern blot	Survival	S100A6	[11]
Secreted proteins/antigens	ELISA	Tumor stage, tumor progression, poor prognosis	YKL-40	[11]
	ELISA	Survival, recurrence	TA90	[11]
	ELISA, RT-PCR	Tumor stage, survival, tumor progression	VEGF	[11]
	ELISA	Survival poor prognosis	MIA	[11
	RT-PCR	Tumor progression	MAGE	[11]
	ELISA	Tumor burden	VEGF-C, VEGFR-3	[11]
	RT-PCR	Tumor stage	MART-1	[11]
	IP	Survival tumor progression	C-reactive protein	[11]
	IHC, TMA	*Breslow index*, survival, poor prognosis	Osteopontin	[11]
	ELISA	Tumor progression	CYT-MAA	[11]
	ELISA	Survival	sICAM, sVCAM	[11]
	IHC, ELISA	Poor prognosis, tumor progression	Galectin-3	[11]
	IHC, ELISA	Tumor stage, tumor progression, overall survival	CEACAM	[11]
Enzymes	HPLC	Overall survival	IDO	[23]
	RT-PCR, nested RT-PCR	Poor prognosis, survival rate, overall survival	Tyrosinase	[11]
	IHC	Disease-free survival	MMP-1, MMP-3	[11]
	Cytomorphology, IHC	Overall survival	CD10	[24, 25]
	IHC	Disease	Cathepsin K	[26]
	ELISA	Disease, poor prognosis	MMP-9	[11, 27]
	ELISA	Disease-free and overall survival	TIMP-1	[27]
	IHC	*Breslow index*, tumor progression	Cox-2	[11]
	IHC	Overall survival	MMP-12	[28]
	Photometric assay, meta-analysis#	Prognosis, tumor stage, survival rate	LDH	[11]
	IHC	Tumor progression	MT1-MMP	[29]
	IHC	Overall survival	Legumain	[30]
	TMA, IHC	Tumor progression	MMP-2	[31, 32]
	IHC	Progression-free survival	MMP-23	[33]

RT-PCR: reverse transcription-polymerase chain reaction; TIMP: tissue inhibitors of metalloproteinase.

## Data Availability

Data sharing does not apply to this article as no datasets were generated or analysed during the current study.

## Additional Points

*Authorship*. The named author meets the International Committee of Medical Journal Editors (ICMJE) criteria for authorship of this article, takes responsibility for the integrity of the work, and has given her approval for this version to be published.
